# Global health inequities in retinoblastoma: a 1990–2021 analysis across socio-demographic index regions

**DOI:** 10.3389/fpubh.2025.1513526

**Published:** 2025-02-21

**Authors:** Xi Li, Ying Chang, Xiaoyu Zhao, Xi Liu, Ying Zhang, Xuan Wang, Junhong Li

**Affiliations:** ^1^Shanxi Eye Hospital Affiliated to Shanxi Medical University, Taiyuan, China; ^2^Department of Ophthalmology, First Hospital of Shanxi Medical University, Taiyuan, China; ^3^Eye Institute, Affiliated Hospital of Nantong University, Nantong, China; ^4^Department of Ultrasound, Children’s Hospital of Shanxi and Women Health Center of Shanxi, Taiyuan, China

**Keywords:** retinoblastoma, health inequality, global burden of disease study, demographic index, slope index, concentration index

## Abstract

**Objective:**

To assess health inequities associated with retinoblastoma across various Socio-Demographic Index (SDI) regions and evaluate whether these inequities have decreased from 1990 to 2021, with the aim of enhancing awareness and guiding government policies.

**Design:**

Population-based demographic analysis.

**Participants:**

Children diagnosed with retinoblastoma from 204 countries and territories.

**Methods:**

The estimates and their 95% uncertainty interval (UI) for disability-adjusted life-years (DALYs) of retinoblastoma were extracted from Global Burden of Disease study (GBD) 2021. The age-standardized DALYs and the average annual percentage change (AAPC) were evaluated.

**Main measures:**

The Slope Index of Inequality (SII) and concentration index were computed to quantify the absolute and relative cross-national health inequality.

**Results:**

All SDI regions and the majority of countries experienced a significant decline in age-standardized DALYs from 1990 to 2021. The decrease was more rapid in middle to high SDI regions than in low to low-middle SDI regions. Globally, the 2–4 years age group had the highest DALYs rate, consistent with trends in low to middle SDI regions. In contrast, the highest DALYs rate in high and high-middle SDI regions was found in the 12–23 months age group. The SII was −40.81 (95% CI −36.04 to −45.58) DALYs per 100,000 population in 1990 and − 30.32 (95% CI −27.18 to −33.47) DALYs per 100,000 population in 2021. The concentration index increased from −0.37 (95% CI −0.46 to −0.28) in 1990 to −0.45 (95% CI −0.53 to −0.36) in 2021, although this increase did not reach statistical significance (*p* = 0.256).

**Conclusion:**

Despite advancements in retinoblastoma management, the overall burden of the disease-related DALY remains disproportionately concentrated in poorer populations. The health inequalities are persisting and widening. This underscores the limitations of current efforts. Until progress benefits everyone, the vision of equitable healthcare remains imperfect.

## Introduction

Retinoblastoma is the most common primary eye malignancy in children, affecting over 8,000 children globally each year (1). This childhood cancer is highly curable when detected early, making it a critical indicator of healthcare efficacy across different regions (2, 3). Despite its treatability, significant global disparities exist in the detection and treatment outcomes of retinoblastoma, primarily influenced by socioeconomic factors (4–9). Advances in screening, detection, and management in high-income countries have led to dramatic improvements in disease outcomes, with nearly all children in these regions surviving (10, 11). However, such advancements have not been mirrored in many lower-income settings, where patients often present with advanced disease, and some even have distant metastasis at the time of initial diagnosis (12–14).

Efforts to address inequities in retinoblastoma treatment have been ongoing for decades (15–18). Various countries and international organizations have implemented initiatives aimed at improving early detection and access to treatment. These initiatives emphasize international cooperation, standardizing treatment protocols, and leveraging technology to improve outcomes globally (7, 15–23). Evaluating their impact on reducing inequities is crucial for providing policymakers with essential decision-making information. However, few studies have examined trends in these inequities, particularly using globe salvage or eye preservation as comparative indicators between regions (8). These measures do not comprehensively represent the treatment outcomes of retinoblastoma, as the primary goal is to ensure disease-free survival for the child, with globe preservation being secondary (8, 24). Thus, a unified, global analysis of retinoblastoma burden and health disparities is urgently needed to enhance awareness and guide government policies.

The Global Burden of Diseases, Injuries, and Risk Factors Study (GBD) 2021 has, for the first time, documented the burden of retinoblastoma (25). This study uses disability-adjusted life years (DALYs) as a key metric to provide a comprehensive estimation of disease burden. DALYs combine years of life lost (YLL) due to retinoblastoma and years lived with disability (YLD) resulting from retinoblastoma, offering a more scientifically robust measure than globe salvage rates.

As recommended by the World Health Organization (WHO), the slope index of inequality (SII) and the concentration index are the two most common measures used to summarize health inequality across subgroups (26). Both measures’ strengths lie in their population-weighted calculation, yielding a single number that accurately reflects inequality across all subgroups, accounting for the size of each subgroup (26).

In this study, we utilized data from the GBD 2021 database to (1) assess health inequities associated with retinoblastoma across various Socio-Demographic Index (SDI) regions and (2) analyze trends in these health disparities using the SII and concentration index from 1990 to 2021. This analysis seeks to provide valuable insights for policymakers to develop targeted strategies to mitigate health disparities and improve outcomes for children with retinoblastoma worldwide.

## Materials and methods

### Data sources

This analysis is based on data from the 2021 Global Burden of Disease (GBD) study, coordinated by the Institute for Health Metrics and Evaluation (IHME). The 2021 GBD study provides the largest and most recent comparative estimates of global disease burden, including incidence, prevalence, and disability-adjusted life-years (DALYs) for 371 diseases and injuries, at both country and subnational levels from 1990 to 2021. The methodology employed in the GBD 2021 has been extensively documented in prior studies (25, 27).

We extracted estimates and 95% uncertainty intervals (UI) for DALYs to measure the burden of retinoblastoma [GBD cause code: B.10.1; International Classification of Diseases (ICD)-10 code: C69.2-C69.22]. The Socio-Demographic Index (SDI) was used as an indicator of a nation’s or region’s socioeconomic status, which correlates significantly with overall health indicators. The SDI considers factors such as the fertility rate of women under 25, average educational attainment, and lag-distributed income per capita in each country or territory.

### Burden description

The GBD 2021 provided non-zero burden estimates for retinoblastoma in children under 9 years old, while the default age-standardized DALY rates were estimated across the entire age range from 0 to 99+ years. To facilitate comparisons of disease burden across different years and regions, we estimated age-standardized DALY rates and corresponding 95% confidence intervals (CIs) for children aged 0–9 years, using crude rates for two age subgroups (0–4 years and 5–9 years) and the world standard population. We examined the relationship between the burden of retinoblastoma-related DALY in 2021 and the SDI at the national level using Spearman’s rank correlation. We also investigated the distribution of DALY rates across age groups (<1 year, 12–23 months, 2–4 years, and 5–9 years) in different SDI regions to determine which age groups experience the highest burden of retinoblastoma. We employed Joinpoint regression to estimate average annual percentage change (AAPC) as a measure of temporal trends (28). AAPC quantify the average annual increase or decrease of a specific variable over a defined period. For our study period from 1990 to 2021, AAPC were calculated from the weighted average of slope coefficients obtained from the Joinpoint regression model.

### Measurement of health inequalities

Total DALYs and age-standardized DALY rates were extracted for the analysis of inequality. The Slope Index of Inequality (SII) and the concentration index were used to measure absolute and relative income-related inequality across countries, respectively. The SII was determined by performing a regression analysis on national DALY rates against a relative position scale associated with the SDI. This scale was defined by the midpoint of the cumulative population distribution ranked by SDI. Heteroscedasticity was adjusted using iteratively reweighted least squares. The concentration index was obtained by numerically integrating the area under the Lorenz concentration curve, which was constructed using the cumulative proportion of DALYs and the cumulative relative distribution of the population ranked by SDI. The concentration index was calculated as twice the area between the 45° diagonal line (line of equality) and the Lorenz curve.

AAPCs were calculated using the Joinpoint Regression Program (version 5.0.2). The SII and concentration index were computed and visualized using Stata MP16.0 and R software V.4.3.3. All other analyses and visualizations were executed using R software V.4.3.3.

## Results

### Burden of retinoblastoma

Globally, the DALYs for retinoblastoma decreased from 276,066 in 1990 to 243,204 in 2021. The age-standardized DALYs for retinoblastoma dropped from 23 per 100,000 population in 1990 to 18.64 per 100,000 population in 2021, with an average annual decrease of −0.67%. In both 1990 and 2021, the highest age-standardized DALYs were in the low SDI region, with 56.69 per 100,000 population in 1990 and 39.38 per 100,000 population in 2021. Conversely, the high SDI region recorded the lowest age-standardized DALYs for both years, at 3.19 per 100,000 population in 1990 and 1.32 per 100,000 population in 2021 (Table 1). All SDI regions experienced a significant decline in age-standardized DALYs from 1990 to 2021 (all AAPC *p*-values <0.001). The decrease was more rapid in high-middle (AAPC = −3.2%) and high SDI (AAPC = −2.9%) regions compared to middle (AAPC = −2.09%), low (AAPC = −1.14%), and middle-low SDI (AAPC = −0.99%) regions (Table 1).

**Table 1:** Global and regional burden of retinoblastoma and the AAPC of age-standardized DALYs rate, 1990–2021.

	DALYs Number		Age-standardized DALYs Rate		
	1990 (95%UI)	2021 (95%UI)	1990 (95%UI)	2021 (95%UI)	AAPC (95%CI)
Global	279,066 (157,364, 368,779)	243,204 (147,119, 333,465)	23 (12.97, 30.4)	18.67 (11.3, 25.61)	−0.67 (−0.71, −0.62)*
SDI					
High	3,920 (3,219, 4,737)	1,445 (1,129, 1819)	3.19 (2.62, 3.86)	1.32 (1.03, 1.66)	−2.9 (−3.21, −2.71) *
High-middle	18,894 (10,552, 29,433)	5,661 (2,805, 8,482)	10.34 (5.77, 16.12)	3.97 (1.95, 5.96)	−3.2 (−3.51, −2.99) *
Middle	57,028 (33,187, 76,499)	27,522 (15,589, 37,596)	14.49 (8.43, 19.44)	7.73 (4.37, 10.57)	−2.09 (−2.23, −2.01) *
Low-middle	99,163 (53,321, 141,906)	80,918 (48,450, 114,807)	29.3 (15.75, 41.95)	21.38 (12.8, 30.32)	−0.99 (−1.05, −0.95) *
Low	99,940 (55,531, 138,761)	127,543 (76,375, 186,075)	56.69 (31.48, 78.8)	39.38 (23.58, 57.45)	−1.14 (−1.17, −1.11) *

DALYs, disability-adjusted life-years; AAPC, average annual percentage change; UI, uncertainty interval; CI, confidence interval; SDI, sociodemographic index. *AAPC *p*-values < 0.001.

**Figure 1:**
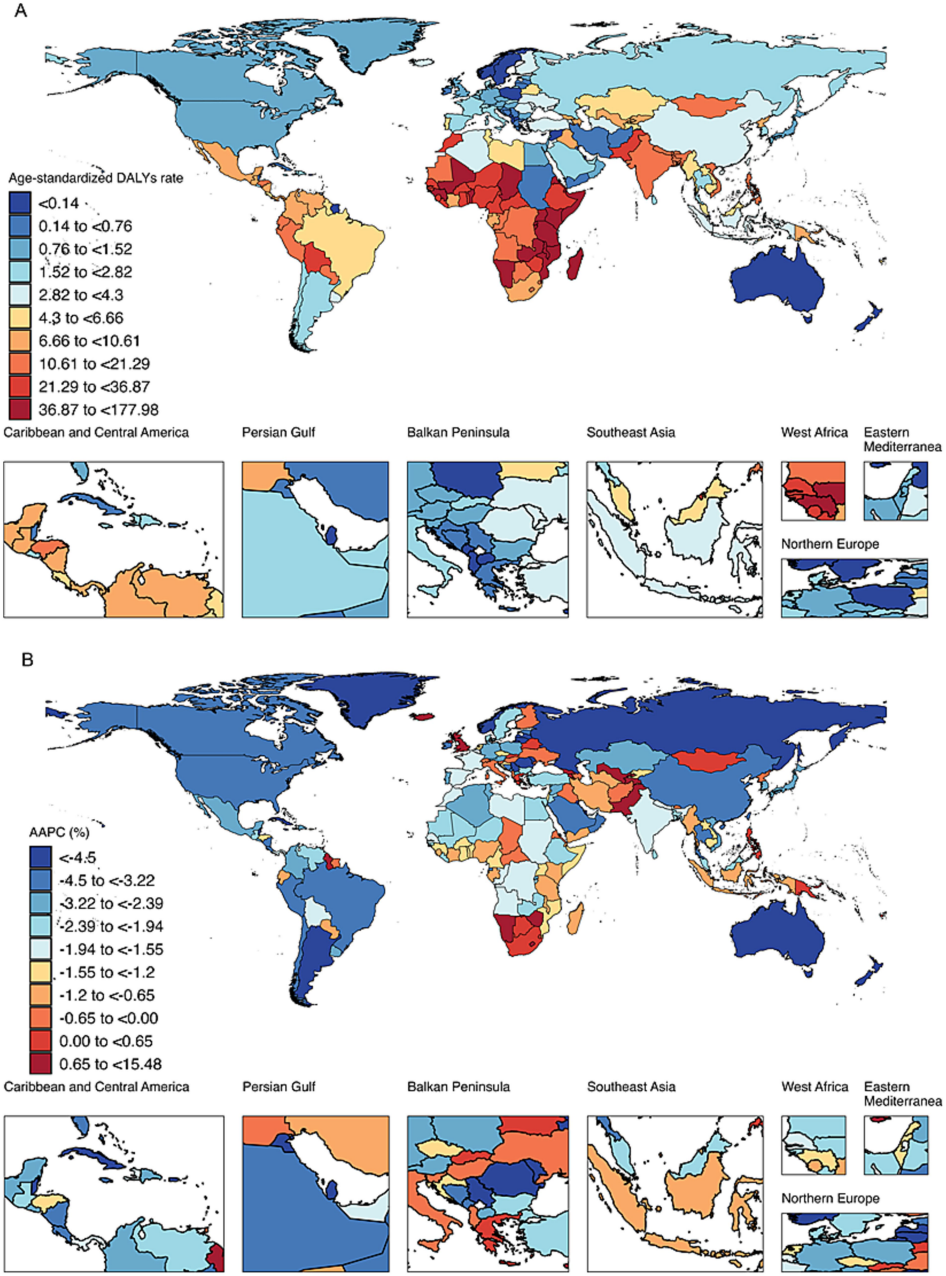
**(A)** Spatial distribution of age-standardized DALYs for retinoblastoma in 2021. DALYs, disability-adjusted life years. **(B)** Spatial distribution of average annual percentage change of age-standardized DALYs for retinoblastoma from 1990 to 2021. DALYs, disability-adjusted life years. AAPC, average annual percentage change.

At the national level, among the top 10 countries with the highest age-standardized DALYs for retinoblastoma in 2021, Tokelau had the highest value at 177.98. The remaining nine countries were in Africa: Malawi (177.15), Kenya (159.78), Eritrea (97.80), Mozambique (94.27), Comoros (84.03), Tanzania (77.22), Djibouti (69.25), Madagascar (67.75), and Somalia (63.85). In contrast, wealthy island or peninsular nations, such as Seychelles (<0.001), Saint Kitts and Nevis (0.002), Qatar (0.003), and Bermuda (0.01), exhibited the lowest age-standardized DALYs (Figure 1A).

From 1990 to 2021, the majority of countries (165 out of 204) exhibited a decreasing trend in age-standardized DALYs for retinoblastoma. Among these, Cuba showed the most significant decline with an AAPC of −11.50%, followed by Kuwait (AAPC = −10.59%) and Lithuania (AAPC = −10.14%). Among the countries exhibiting an increasing trend, Tokelau experienced the most rapid rise with an AAPC of 15.48%, followed by Armenia (AAPC = 9.68%) and Georgia (AAPC = 6.74%; Figure 1B).

**Figure 2:**
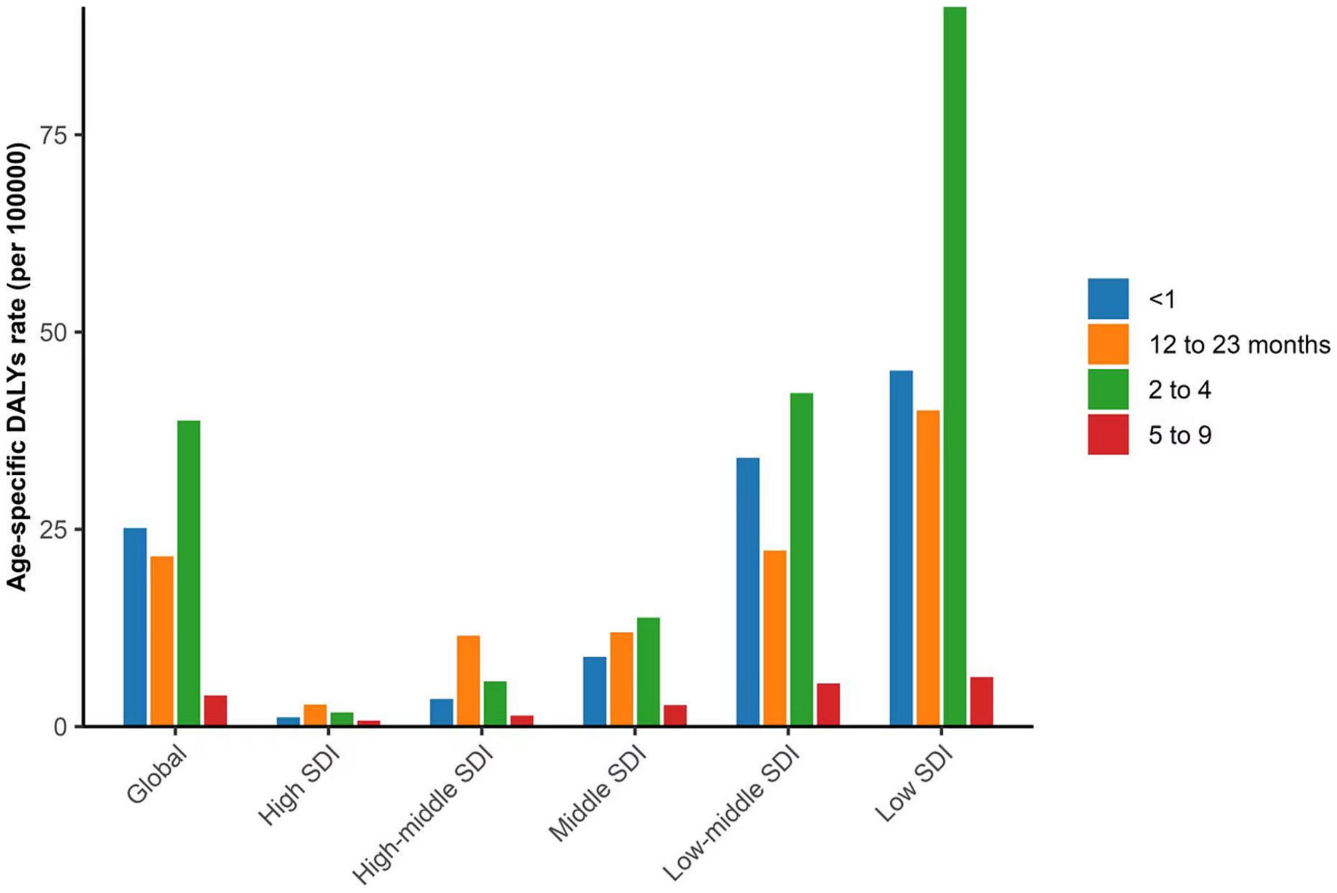
Age-specific DALYs Rate across different SDI regions in 2021. DALYs, disability-adjusted life-years; SDI, Sociodemographic Index.

At national level in 2021, the association between the SDI and DALYs revealed a significant negative correlation (Spearman’s *ρ* = −0.71, *p* < 0.001). Globally, the 2–4 years age group had the highest DALY rate, a trend consistent in low, low-middle, and middle SDI regions. In contrast, the highest DALY rate in high and high-middle SDI regions was found in the 12–23 months age group (Figure 2).

### Cross-national health inequality

**Figure 3:**
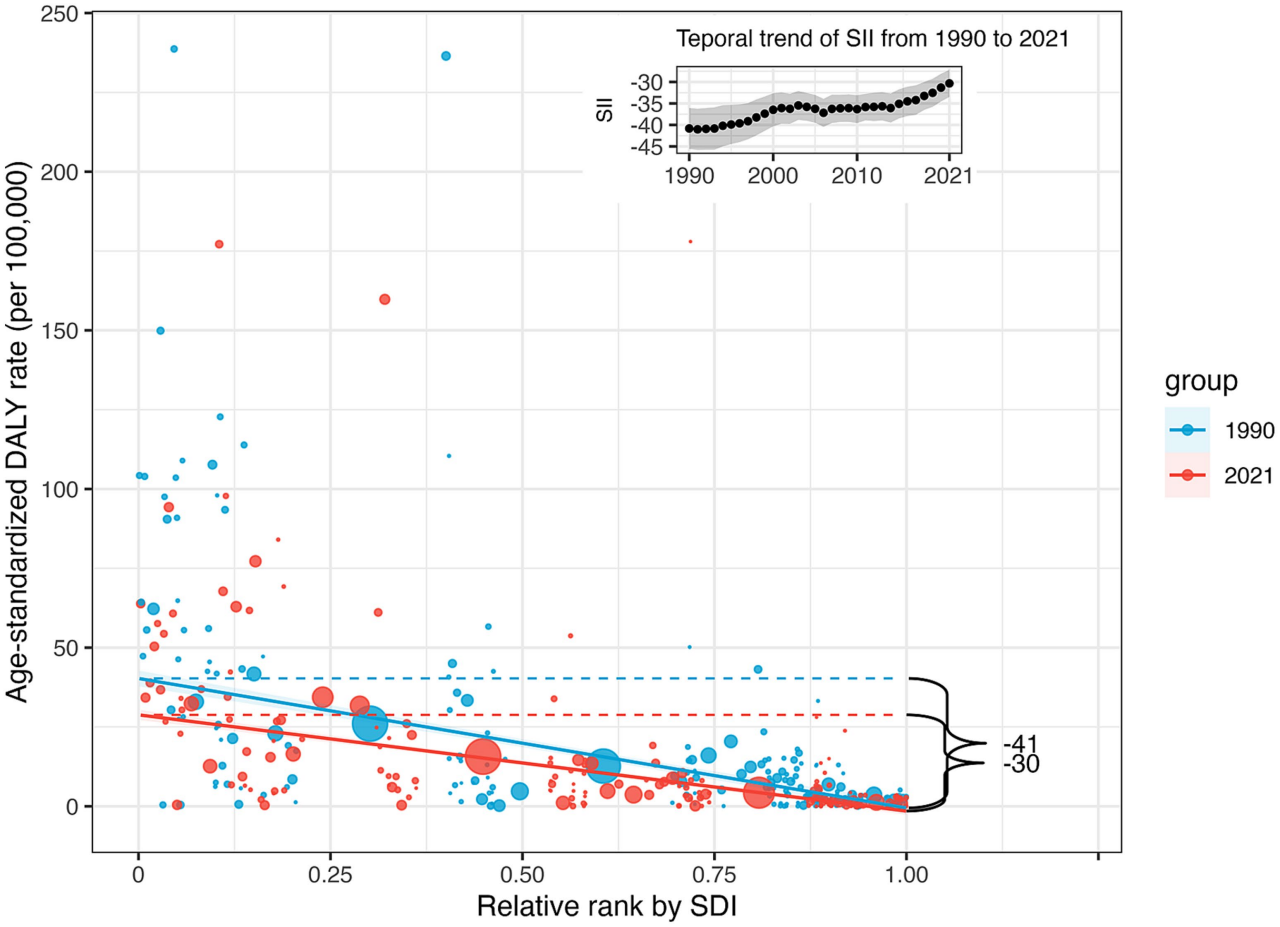
Absolute SDI-related health inequality, presented using regression lines, for age-standardized DALYs rate of retinoblastoma across 204 counties and territories, 1990 vs. 2021. And temporal trend of Slope Index of Inequality from 1990 to 2021. DALYs, disability-adjusted life-years; SDI, Sociodemographic Index. SII, Slope Index of Inequality.

**Figure 4:**
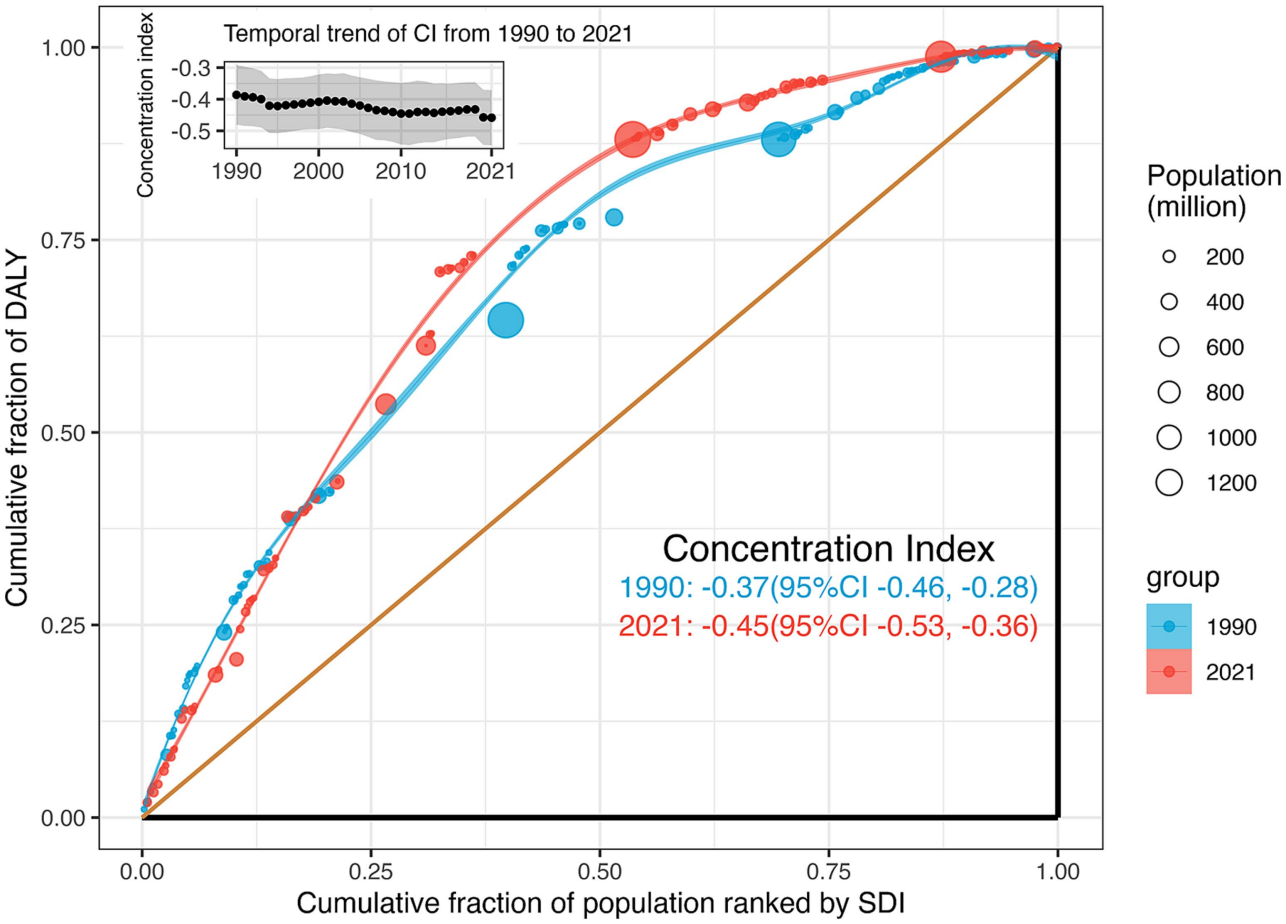
Relative SDI-related health inequality, presented using concentration curves, for age-standardized DALYs rate of retinoblastoma across 204 counties and territories, 1990 vs. 2021. And temporal trend of the concentration index from 1990 to 2021. DALYs, disability-adjusted life-years; SDI, Sociodemographic Index.

Significant absolute and relative SDI-related inequalities in the burden of retinoblastoma were observed, with a disproportionately higher burden shouldered by countries with lower SDI (Figure 3). The SII was −40.81 (95% CI −36.04 to −45.58) DALYs per 100,000 population in 1990 and − 30.32 (95% CI −27.18 to −33.47) DALYs per 100,000 population in 2021. This decline indicates that the absolute inequality in the age-standardized burden of retinoblastoma between high and low SDI countries narrowed during this period (Figure 3). Relative inequality analysis showed that the absolute values for the concentration index remained above 0.37 from 1990 to 2021, indicating a reasonably high level of relative inequality. The values exhibited an increasing trend between 1990 (−0.37, 95% CI −0.46 to −0.28) and 2021 (−0.45, 95% CI −0.53 to −0.36), although this increase did not reach statistical significance (diff = −0.07, 95% CI −0.2 to 0.05, *p* = 0.256; Figure 4).

## Discussion

This secondary analysis of data from the GBD 2021 offers a comparative overview of the global burden of retinoblastoma, socio-economic-related inequalities, and temporal trends from 1990 to 2021. Our study found that, despite a reduction in the global age-standardized burden of retinoblastoma, health inequalities related to this disease remain significant and persistent.

Our study confirmed a global decline in the burden of retinoblastoma-related DALY from 1990 to 2021. This decline is likely due to the establishment of specialized referral centers, advancements in genetic understanding, and the introduction of chemotherapy in recent decades (29). Despite this overall global decrease, the age-standardized DALYs and the extent of this decline varied significantly at the national level. Regions with middle to high SDI experienced more pronounced decreases compared to low and middle-low SDI regions, reflecting health inequities across different socioeconomic contexts.

We found that DALY rates were highest in the 12–23 months age group in high and middle-high SDI regions, whereas in regions with lower SDI, DALY rates peaked in the 2–4-year age group. These findings align with previous research indicating that the age at onset of retinoblastoma is younger in high-income regions compared to low-income regions (4, 7). The Global Retinoblastoma Study Group previously reported that the median age at diagnosis of retinoblastoma worldwide is 24 months: 31 months in low-income countries and 14 months in high-income countries (4).

Previous studies by the Global Retinoblastoma Study Group (4), the American Joint Committee on Cancer Ophthalmic Oncology Task Force (7), and meta-analyses by Wong et al. (8) have focused on the relationships between global retinoblastoma presentation and income level. These studies demonstrated that higher income levels are associated with increased overall survival, better globe salvage rates, younger age at diagnosis, and lower proportions of locally advanced disease and distant metastasis. However, these studies typically employed simple measures of inequality, comparing health outcomes between selected regions. However, “complex measures” recommended by the WHO offer a more comprehensive view of inequality across all regions or countries (26). These measures can provide a deeper understanding of disparities in health outcomes related to retinoblastoma.

In this study, we used two WHO-recommended measures: the SII and the concentration index. These measures rely on the cross-national health gradient and the SDI ordering. By weighting population size, they provide a comprehensive description of inequality across all subgroups and track trends over time. This approach is crucial for monitoring health inequalities and evaluating the impact of social policies. Effective social policies that reduce poverty, enhance educational opportunities, or create jobs can decrease the size of disadvantaged subgroups (26). Therefore, assessing the impact of such policies on health inequality is essential for policymakers.

By calculating the SII and the concentration index, we found that the burden of retinoblastoma was negatively associated with socioeconomic level. From 1990 to 2021, this inequality saw a slight reduction in absolute terms (SII) but remained consistently high in relative terms (concentration index). To our knowledge, these findings have not been previously reported.

The disparity in trends between absolute and relative eye health inequality can be attributed to differences in their computational methods (30). Absolute inequality, quantified by the SII, represents the absolute difference in predicted values of a health indicator between the highest and lowest SDI levels, considering the entire SDI distribution through a regression model. As SII is influenced by measurement scales, a reduction in SII alongside decreases in the age-standardized DALY rate is anticipated. Conversely, relative inequality, measured by the concentration index, assesses proportional health differences among socioeconomic groups. Negative values indicate a concentration among the disadvantaged, and positive values among the advantaged. Despite a theoretical range of ±1, practical values rarely exceed 0.5, with 0.2 to 0.3 indicating high relative inequality (26). Since 1990, the concentration index for retinoblastoma has consistently exceeded −0.37, reaching its peak at −0.45 in 2021, indicating a persistently high level of relative inequality and slight worsening over time. Thus, our findings underscore the ongoing severity of health inequality in retinoblastoma.

According to the WHO, there are significant disparities in childhood cancer outcomes both between and within countries, with socioeconomic status being a major driver of these inequities (31). Identified by the WHO as a “tracer cancer, “retinoblastoma serves as a marker for global healthcare transformation and progress (3, 23). Despite ongoing efforts to improve retinoblastoma outcomes globally, our study reveals the persistent and severe nature of health inequalities in this disease, necessitating further reflection and action. This requires collective investment, particularly in addressing the current gaps in research funding. Our findings highlight the urgent need for targeted interventions to improve affordable early detection strategies and enhance treatment accessibility in underserved populations. Standardizing treatment protocols and leveraging technological advancements are crucial to narrowing health inequalities and improving survival rates for children with retinoblastoma worldwide. These efforts must be supported by global health policies that prioritize equitable resource allocation, particularly in low-income regions, where increased investment in healthcare infrastructure is essential for ensuring timely access to diagnostic and treatment services. Additionally, fostering international collaborations to share research, expertise, and best practices in retinoblastoma treatment, while advocating for affordable diagnostic technologies, is key to advancing global health outcomes. Policy recommendations should focus on establishing screening programs and ensuring access to services, particularly in low- and middle-SDI regions, to move closer to achieving equitable access to treatment and care for all. Until progress benefits everyone, the vision of equitable healthcare remains imperfect. International collaborations.

This secondary analysis has several strengths. It uses the latest data and standard methods for a comprehensive global assessment of health equity. The insights provided by this study are difficult to obtain from isolated regional studies. However, the study has limitations. First, the GBD data source and methodology have certain weaknesses. Limited data quality may introduce biases into the analysis. Additionally, the study is constrained by the ecological fallacy since all conclusions are based on national-level data rather than individual-level data. Second, this study focuses on analyzing the outcomes of past global efforts to improve health inequities related to retinoblastoma. It does not evaluate the impact of specific policies on these inequities, which is crucial for future policy development. Finally, although the WHO recommends using the concentration index, it is more susceptible to being influenced by changes in the SDI of populous countries. Future analyses should consider these factors to ensure a balanced assessment.

## Conclusion

In conclusion, despite advancements in retinoblastoma management, the overall burden of the disease-related DALY remains disproportionately concentrated in poorer populations. Furthermore, this health inequality has not significantly improved over the past few decades, underscoring the limitations of current efforts. This persistent disparity highlights the urgent need for targeted interventions focused on underserved communities to achieve the goal of eliminating deaths from this rare yet curable cancer and enhancing the quality of life for affected individuals.

## Data Availability

The datasets presented in this study can be found in online repositories. The names of the repository/repositories and accession number(s) can be found in the article/supplementary material.
